# Supplementary material for: Development of the Sterile Insect Technique to control the dengue vector Aedes aegypti (Linnaeus) in Sri Lanka
Source: PLoS One. 2022 Apr 4;17(4):e0265244. doi: 10.1371/journal.pone.0265244 (PMC8979456; doi:10.1371/journal.pone.0265244)
Supplement: S1 Appendix — (PDF) [file pone.0265244.s001.pdf]

```
> ####       Survival coxme data Sri Lanka   ##################
> ################################################################
>
> #######------------------- male mosquito survival--------------------------------
--
>
> library(readr)
> data <- read_delim("statsmaiga/Srilanka/ms/surv data coxme/male_surv.csv",
+                          ";", escape_double = FALSE, trim_ws = TRUE)

-- Column specification --------------------------------------------------------
cols(
  id = col_double(),
  dose = col_character(),
  time = col_double(),
  replicate = col_character(),
  event = col_double()
)

>
> View(data)
> str(data)
spec_tbl_df[,5] [450 x 5] (S3: spec_tbl_df/tbl_df/tbl/data.frame)
 $ id       : num [1:450] 1 2 3 4 5 6 7 8 9 10 ...
 $ dose     : chr [1:450] "b" "e" "c" "d" ...
 $ time     : num [1:450] 1 1 2 2 2 2 2 2 3 3 ...
 $ replicate: chr [1:450] "R1" "R1" "R1" "R1" ...
 $ event    : num [1:450] 1 1 1 1 1 1 1 1 1 1 ...
 - attr(*, "spec")=
  .. cols(
  ..   id = col_double(),
  ..   dose = col_character(),
  ..   time = col_double(),
  ..   replicate = col_character(),
  ..   event = col_double()
  .. )
> data <- transform(data,dose=factor(dose),
+                   replicate=factor(replicate))
>
> str(data)
'data.frame':	450 obs. of  5 variables:
 $ id       : num  1 2 3 4 5 6 7 8 9 10 ...
 $ dose     : Factor w/ 6 levels "a","b","c","d",..: 2 5 3 4 4 5 6 6 3 4 ...
 $ time     : num  1 1 2 2 2 2 2 2 3 3 ...
 $ replicate: Factor w/ 3 levels "R1","R2","R3": 1 1 1 1 3 2 1 3 2 2 ...
 $ event    : num  1 1 1 1 1 1 1 1 1 1 ...
> summary(data)
       id         dose        time       replicate     event       
 Min.   :  1.0   a:75   Min.   : 1.00   R1:150    Min.   :0.0000  
 1st Qu.:113.2   b:75   1st Qu.:13.00   R2:150    1st Qu.:1.0000  
 Median :225.5   c:75   Median :17.00   R3:150    Median :1.0000  
 Mean   :225.5   d:75   Mean   :17.22             Mean   :0.9622  
 3rd Qu.:337.8   e:75   3rd Qu.:22.00             3rd Qu.:1.0000  
 Max.   :450.0   f:75   Max.   :32.00             Max.   :1.0000  
> attach(data)
The following objects are masked from data (pos = 3):

    dose, event, id, replicate, time

The following objects are masked from data (pos = 4):

    dose, event, id, replicate, time

The following objects are masked from data (pos = 22):

    dose, event, id, replicate, time

>
```

```
>
> ##### STATISTICS########
>
> ########  ---- coxme model---------------
>
> library(coxme)
> data$dose<-relevel(data$dose, ref = "a", data) ##control as reference-##
> mod <- coxme(Surv(time,event)~dose + ( 1|replicate), data=data)
> summary(mod)
Cox mixed-effects model fit by maximum likelihood
  Data: data
  events, n = 433, 450
  Iterations= 5 28
                    NULL Integrated    Fitted
Log-likelihood -2212.598  -2204.303 -2203.065

                  Chisq   df          p  AIC    BIC
Integrated loglik 16.59 6.00 0.0109120 4.59 -19.83
 Penalized loglik 19.07 6.03 0.0041383 7.01 -17.53

Model:  Surv(time, event) ~ dose + (1 | replicate)
Fixed coefficients
             coef exp(coef)  se(coef)     z       p
doseb -0.01498035 0.9851313 0.1681494 -0.09 0.93000
dosec  0.10873798 1.1148702 0.1660055  0.66 0.51000
dosed  0.28681271 1.3321747 0.1675737  1.71 0.08700
dosee  0.28533108 1.3302024 0.1695693  1.68 0.09200
dosef  0.57443218 1.7761217 0.1699610  3.38 0.00073

Random effects
 Group     Variable  Std Dev     Variance
 replicate Intercept 0.086283073 0.007444769
>
> #----Graphs-------#
> library(tidyverse)
> library(survival)
> library(survminer)
> fit<-survfit(Surv(time,event)~dose, data = data)
>
> class(fit)
[1] "survfit"
> summary(fit)
Call: survfit(formula = Surv(time, event) ~ dose, data = data)

                dose=a
 time n.risk n.event survival std.err lower 95% CI upper 95% CI
    6     75       2   0.9733  0.0186       0.9375        1.000
    7     73       1   0.9600  0.0226       0.9167        1.000
    8     72       1   0.9467  0.0259       0.8972        0.999
    9     71       2   0.9200  0.0313       0.8606        0.983
   10     69       3   0.8800  0.0375       0.8094        0.957
   13     66       3   0.8400  0.0423       0.7610        0.927
   14     63       5   0.7733  0.0483       0.6842        0.874
   15     58       2   0.7467  0.0502       0.6544        0.852
   16     55       7   0.6516  0.0552       0.5520        0.769
   17     48       4   0.5973  0.0569       0.4956        0.720
   18     44       7   0.5023  0.0581       0.4004        0.630
   19     36       5   0.4325  0.0578       0.3329        0.562
   20     31       6   0.3488  0.0558       0.2549        0.477
   21     25       5   0.2791  0.0526       0.1928        0.404
   22     20       4   0.2232  0.0490       0.1453        0.343
   23     16       2   0.1953  0.0466       0.1223        0.312
   24     14       2   0.1674  0.0440       0.1001        0.280
   25     12       1   0.1535  0.0425       0.0893        0.264
   26     11       1   0.1395  0.0408       0.0786        0.248
   28      9       2   0.1085  0.0372       0.0555        0.212
   29      7       2   0.0775  0.0324       0.0342        0.176
   30      5       2   0.0465  0.0258       0.0157        0.138
```

31	3	3	0.0000	NaN	NA	NA

dose=b

time	n.risk	n.event	survival	std.err	lower 95% CI	upper 95% CI
1	75	1	0.9867	0.0132	0.96105	1.000
4	74	1	0.9733	0.0186	0.93755	1.000
5	73	1	0.9600	0.0226	0.91666	1.000
7	72	1	0.9467	0.0259	0.89716	0.999
8	71	3	0.9067	0.0336	0.84316	0.975
9	68	5	0.8400	0.0423	0.76100	0.927
10	63	3	0.8000	0.0462	0.71441	0.896
11	59	2	0.7729	0.0484	0.68354	0.874
13	57	3	0.7322	0.0513	0.63831	0.840
14	54	5	0.6644	0.0548	0.56530	0.781
15	48	3	0.6229	0.0563	0.52169	0.744
16	44	3	0.5804	0.0576	0.47784	0.705
17	41	3	0.5379	0.0584	0.43490	0.665
18	38	6	0.4530	0.0585	0.35163	0.584
19	32	2	0.4247	0.0582	0.32464	0.556
20	30	3	0.3822	0.0573	0.28488	0.513
21	27	5	0.3114	0.0548	0.22066	0.440
22	22	3	0.2690	0.0525	0.18348	0.394
23	19	1	0.2548	0.0516	0.17134	0.379
24	18	1	0.2407	0.0506	0.15933	0.364
25	17	2	0.2123	0.0485	0.13574	0.332
26	15	1	0.1982	0.0473	0.12418	0.316
27	13	3	0.1525	0.0431	0.08759	0.265
28	10	3	0.1067	0.0374	0.05369	0.212
29	7	1	0.0915	0.0350	0.04319	0.194
30	6	3	0.0457	0.0256	0.01527	0.137
31	3	2	0.0152	0.0151	0.00219	0.106
32	1	1	0.0000	NaN	NA	NA

dose=c

time	n.risk	n.event	survival	std.err	lower 95% CI	upper 95% CI
2	75	1	0.9867	0.0132	0.9610	1.000
3	74	1	0.9733	0.0186	0.9375	1.000
6	73	1	0.9600	0.0226	0.9167	1.000
7	72	1	0.9467	0.0259	0.8972	0.999
8	71	2	0.9200	0.0313	0.8606	0.983
9	69	2	0.8933	0.0356	0.8261	0.966
10	67	3	0.8533	0.0409	0.7769	0.937
11	64	2	0.8267	0.0437	0.7453	0.917
12	62	2	0.8000	0.0462	0.7144	0.896
13	60	4	0.7467	0.0502	0.6544	0.852
14	56	5	0.6800	0.0539	0.5822	0.794
15	51	4	0.6267	0.0559	0.5262	0.746
16	47	4	0.5733	0.0571	0.4716	0.697
17	43	6	0.4933	0.0577	0.3922	0.621
18	37	7	0.4000	0.0566	0.3032	0.528
19	30	4	0.3467	0.0550	0.2541	0.473
20	26	2	0.3200	0.0539	0.2301	0.445
22	24	1	0.3067	0.0532	0.2182	0.431
23	23	3	0.2667	0.0511	0.1832	0.388
24	20	5	0.2000	0.0462	0.1272	0.314
25	15	3	0.1600	0.0423	0.0953	0.269
27	12	1	0.1467	0.0409	0.0850	0.253
28	11	2	0.1200	0.0375	0.0650	0.221
29	9	4	0.0667	0.0288	0.0286	0.155
30	5	5	0.0000	NaN	NA	NA

dose=d

time	n.risk	n.event	survival	std.err	lower 95% CI	upper 95% CI
2	75	2	0.9733	0.0186	0.93755	1.000
3	73	1	0.9600	0.0226	0.91666	1.000
10	72	1	0.9467	0.0259	0.89716	0.999
11	71	6	0.8667	0.0393	0.79305	0.947
12	65	5	0.8000	0.0462	0.71441	0.896

13	60	7	0.7067	0.0526	0.61079	0.818
14	52	7	0.6115	0.0565	0.51030	0.733
15	45	3	0.5708	0.0574	0.46866	0.695
16	42	4	0.5164	0.0580	0.41436	0.644
17	38	7	0.4213	0.0574	0.32255	0.550
18	31	4	0.3669	0.0561	0.27198	0.495
19	27	5	0.2990	0.0533	0.21083	0.424
20	22	3	0.2582	0.0509	0.17539	0.380
21	19	2	0.2310	0.0491	0.15235	0.350
22	17	1	0.2174	0.0480	0.14102	0.335
23	15	3	0.1739	0.0445	0.10535	0.287
25	12	1	0.1595	0.0431	0.09388	0.271
26	11	2	0.1305	0.0398	0.07171	0.237
27	9	1	0.1160	0.0380	0.06105	0.220
28	8	4	0.0580	0.0279	0.02255	0.149
29	4	3	0.0145	0.0144	0.00208	0.101
30	1	1	0.0000	NaN	NA	NA

dose=e

time	n.risk	n.event	survival	std.err	lower 95% CI	upper 95% CI
1	75	1	0.9867	0.0132	0.9610	1.000
2	74	1	0.9733	0.0186	0.9375	1.000
4	73	1	0.9600	0.0226	0.9167	1.000
5	72	2	0.9333	0.0288	0.8786	0.992
6	70	1	0.9200	0.0313	0.8606	0.983
8	69	1	0.9067	0.0336	0.8432	0.975
9	68	1	0.8933	0.0356	0.8261	0.966
10	67	2	0.8667	0.0393	0.7930	0.947
11	65	2	0.8400	0.0423	0.7610	0.927
12	63	3	0.8000	0.0462	0.7144	0.896
13	60	6	0.7200	0.0518	0.6252	0.829
14	53	4	0.6657	0.0546	0.5668	0.782
15	49	5	0.5977	0.0568	0.4961	0.720
16	44	3	0.5570	0.0576	0.4547	0.682
17	40	6	0.4734	0.0582	0.3720	0.602
18	34	6	0.3899	0.0571	0.2927	0.519
19	28	4	0.3342	0.0553	0.2416	0.462
20	23	1	0.3197	0.0548	0.2285	0.447
22	22	1	0.3051	0.0542	0.2155	0.432
23	21	7	0.2034	0.0478	0.1283	0.323
24	13	3	0.1565	0.0438	0.0904	0.271
26	10	2	0.1252	0.0403	0.0667	0.235
27	8	3	0.0782	0.0330	0.0342	0.179
28	5	5	0.0000	NaN	NA	NA

dose=f

time	n.risk	n.event	survival	std.err	lower 95% CI	upper 95% CI
2	75	2	0.9733	0.0186	0.93755	1.000
3	73	3	0.9333	0.0288	0.87855	0.992
4	69	3	0.8928	0.0358	0.82521	0.966
5	66	2	0.8657	0.0395	0.79160	0.947
6	64	1	0.8522	0.0412	0.77521	0.937
7	63	2	0.8251	0.0441	0.74311	0.916
8	61	2	0.7981	0.0466	0.71177	0.895
9	59	1	0.7845	0.0477	0.69636	0.884
10	58	2	0.7575	0.0498	0.66596	0.862
11	55	2	0.7299	0.0516	0.63545	0.838
12	53	4	0.6749	0.0546	0.57591	0.791
13	48	5	0.6046	0.0572	0.50216	0.728
14	43	3	0.5624	0.0582	0.45913	0.689
15	39	4	0.5047	0.0589	0.40144	0.635
16	35	3	0.4614	0.0589	0.35923	0.593
17	32	3	0.4182	0.0585	0.31794	0.550
18	29	7	0.3172	0.0554	0.22525	0.447
19	22	6	0.2307	0.0503	0.15046	0.354
20	16	2	0.2019	0.0480	0.12670	0.322
21	14	1	0.1875	0.0467	0.11507	0.305
22	13	2	0.1586	0.0437	0.09241	0.272

```
   23     11       3   0.1154  0.0383      0.06021        0.221
   24      8       2   0.0865  0.0337      0.04032        0.186
   25      6       1   0.0721  0.0310      0.03103        0.168
   26      5       2   0.0433  0.0244      0.01431        0.131
   27      3       2   0.0144  0.0143      0.00206        0.101
   28      1       1   0.0000     NaN           NA           NA

> summary(fit)$table
       records n.max n.start events   *rmean *se(rmean) median 0.95LCL 0.95UCL
dose=a      75    75      75     72 18.86280  0.7159240     19      17      20
dose=b      75    75      75     71 18.18824  0.8719612     18      16      21
dose=c      75    75      75     75 18.04000  0.8025382     17      16      19
dose=d      75    75      75     73 17.33215  0.7318888     17      15      18
dose=e      75    75      75     71 17.37546  0.7776770     17      15      19
dose=f      75    75      75     71 15.06613  0.7958711     16      14      18
>
> ## GRAPHS WITH CONF INTERV ###
> ggsurvplot(fit, data=data,
+            pval = TRUE, conf.int = TRUE,
+            fun = "pct",
+            risk.table = TRUE, # Add risk table
+            risk.table.col = "strata", # Change risk table color by treatment
+            size = 1,
+            linetype = "strata",# Change line type by treatment
+            surv.median.line = "hv", # Specify median survival
+            ggtheme = theme_bw(), # Change ggplot2 theme
+            legend.labs=c("0", "40", "50", "60","70","80"),
+            legend = "bottom",
+            xlab = "Time (days)",  xlim = c(1,35),break.time.by = 2,
+            ylab = "Overall survival probability ")
>
>
> ## GRAPHS WITHOUT CONF INTERV ###
>
> ggsurvplot(fit, data=data,
+            pval = FALSE, conf.int = FALSE,
+            fun = "pct",
+            size = 1,
+            linetype = "strata",# Change line type by treatment
+            surv.median.line = "hv", # Specify median survival
+            ggtheme = theme_bw(), # Change ggplot2 theme
+            legend.labs=c("0", "40", "50", "60","70","80"),
+            legend = "bottom",
+            xlab = "Time (days)",  xlim = c(1,35),break.time.by = 2,
+            ylab = "Overall survival probability ")
>
>
> library(multcomp)
> dose<-summary(glht(mod,linfct = mcp(dose="Tukey")))
> summary(dose)

         Simultaneous Tests for General Linear Hypotheses

Multiple Comparisons of Means: Tukey Contrasts


Fit: coxme(formula = Surv(time, event) ~ dose + (1 | replicate), data = data)

Linear Hypotheses:
            Estimate Std. Error z value Pr(>|z|)
b - a == 0 -0.014980   0.168149  -0.089  1.00000
c - a == 0  0.108738   0.166005   0.655  0.98664
d - a == 0  0.286813   0.167574   1.712  0.52379
e - a == 0  0.285331   0.169569   1.683  0.54313
f - a == 0  0.574432   0.169961   3.380  0.00946 **
c - b == 0  0.123718   0.167642   0.738  0.97721
d - b == 0  0.301793   0.169585   1.780  0.47889
e - b == 0  0.300311   0.171720   1.749  0.49911
```

```
f - b == 0  0.589413   0.172378   3.419  0.00822 **
d - c == 0  0.178075   0.165055   1.079  0.88994
e - c == 0  0.176593   0.167208   1.056  0.89856
f - c == 0  0.465694   0.167937   2.773  0.06201 .
e - d == 0 -0.001482   0.167095  -0.009  1.00000
f - d == 0  0.287619   0.167454   1.718  0.51990
f - e == 0  0.289101   0.168136   1.719  0.51869
---
Signif. codes:  0 '***' 0.001 '**' 0.01 '*' 0.05 '.' 0.1 ' ' 1
(Adjusted p values reported -- single-step method)

> plot(summary(dose))




> ############################################################
>
>                           ##### surv females###
>
> ###################################################################
>
>
> library(readr)
> data <- read_delim("statsmaiga/Srilanka/ms/surv data coxme/female_surv.csv",
+                     ";", escape_double = FALSE, trim_ws = TRUE)

-- Column specification --------------------------------------------------------
cols(
  id = col_double(),
  dose = col_character(),
  time = col_double(),
  replicate = col_character(),
  event = col_double()
)

>
> View(data)
> str(data)
spec_tbl_df[,5] [450 x 5] (S3: spec_tbl_df/tbl_df/tbl/data.frame)
 $ id       : num [1:450] 1 2 3 4 5 6 7 8 9 10 ...
 $ dose     : chr [1:450] "b" "c" "b" "c" ...
 $ time     : num [1:450] 2 2 3 3 3 3 3 3 4 4 ...
 $ replicate: chr [1:450] "R3" "R1" "R1" "R2" ...
 $ event    : num [1:450] 1 1 1 1 1 1 1 1 1 1 ...
 - attr(*, "spec")=
  .. cols(
  ..   id = col_double(),
  ..   dose = col_character(),
  ..   time = col_double(),
  ..   replicate = col_character(),
  ..   event = col_double()
  .. )
> data <- transform(data,dose=factor(dose),
+                    replicate=factor(replicate))
>
> str(data)
'data.frame':450 obs. of  5 variables:
 $ id       : num  1 2 3 4 5 6 7 8 9 10 ...
 $ dose     : Factor w/ 6 levels "a","b","c","d",..: 2 3 2 3 4 5 6 6 2 5 ...
 $ time     : num  2 2 3 3 3 3 3 3 4 4 ...
 $ replicate: Factor w/ 3 levels "R1","R2","R3": 3 1 1 2 2 3 1 2 2 1 ...
 $ event    : num  1 1 1 1 1 1 1 1 1 1 ...
> summary(data)
       id          dose        time         replicate      event
 Min.   :  1.0   a:75    Min.   : 2.00   R1:150     Min.   :0.0000
 1st Qu.:113.2   b:75    1st Qu.:19.00   R2:150     1st Qu.:1.0000
 Median :225.5   c:75    Median :28.00   R3:150     Median :1.0000
```

```
 Mean   :225.5   d:75   Mean   :27.49             Mean   :0.9444
 3rd Qu.:337.8   e:75   3rd Qu.:34.00             3rd Qu.:1.0000
 Max.   :450.0   f:75   Max.   :52.00             Max.   :1.0000
> attach(data)
The following objects are masked from data (pos = 3):

    dose, event, id, replicate, time

The following objects are masked from data (pos = 4):

    dose, event, id, replicate, time

The following objects are masked from data (pos = 5):

    dose, event, id, replicate, time

The following objects are masked from data (pos = 23):

    dose, event, id, replicate, time

>
>
> ##### STATISTICS########
>
> ########  ---- coxme model---------------
>
> library(coxme)
> data$dose<-relevel(data$dose, ref = "a", data) ##control as reference-##
> mod <- coxme(Surv(time,event)~dose + ( 1|replicate), data=data)
> summary(mod)
Cox mixed-effects model fit by maximum likelihood
  Data: data
  events, n = 425, 450
  Iterations= 5 22
                    NULL Integrated    Fitted
Log-likelihood -2176.626  -2170.136 -2170.111

                  Chisq   df        p  AIC    BIC
Integrated loglik 12.98 6.00 0.043345 0.98 -23.33
 Penalized loglik 13.03 5.03 0.023516 2.98 -17.39

Model:  Surv(time, event) ~ dose + (1 | replicate)
Fixed coefficients
              coef exp(coef)  se(coef)     z      p
doseb -0.008944383 0.9910955 0.1692638 -0.05 0.9600
dosec  0.223303790 1.2502003 0.1715408  1.30 0.1900
dosed  0.337504471 1.4014459 0.1715043  1.97 0.0490
dosee  0.385471487 1.4703074 0.1723619  2.24 0.0250
dosef  0.449230054 1.5671051 0.1738896  2.58 0.0098

Random effects
 Group     Variable  Std Dev      Variance
 replicate Intercept 9.860819e-03 9.723576e-05
>
> #----Graphs-------#
> library(tidyverse)
> library(survival)
> library(survminer)
> fit<-survfit(Surv(time,event)~dose, data = data)
>
> class(fit)
[1] "survfit"
> summary(fit)
Call: survfit(formula = Surv(time, event) ~ dose, data = data)

                dose=a
 time n.risk n.event survival std.err lower 95% CI upper 95% CI
    9     74       1   0.9865  0.0134      0.96053        1.000
```

10	73	1	0.9730	0.0189	0.93672	1.000
12	72	1	0.9595	0.0229	0.91556	1.000
13	71	1	0.9459	0.0263	0.89580	0.999
14	70	1	0.9324	0.0292	0.87696	0.991
16	69	1	0.9189	0.0317	0.85879	0.983
17	68	2	0.8919	0.0361	0.82388	0.966
18	66	3	0.8514	0.0414	0.77404	0.936
19	63	2	0.8243	0.0442	0.74202	0.916
20	61	2	0.7973	0.0467	0.71077	0.894
21	59	3	0.7568	0.0499	0.66505	0.861
22	56	3	0.7162	0.0524	0.62052	0.827
23	53	1	0.7027	0.0531	0.60591	0.815
24	52	5	0.6351	0.0560	0.53440	0.755
25	47	2	0.6081	0.0567	0.50646	0.730
27	45	2	0.5811	0.0574	0.47887	0.705
28	43	3	0.5405	0.0579	0.43813	0.667
29	40	7	0.4459	0.0578	0.34593	0.575
30	33	3	0.4054	0.0571	0.30765	0.534
31	29	2	0.3774	0.0565	0.28154	0.506
32	27	4	0.3215	0.0546	0.23053	0.448
33	23	2	0.2936	0.0533	0.20568	0.419
34	21	2	0.2656	0.0518	0.18129	0.389
35	19	1	0.2516	0.0509	0.16929	0.374
37	18	2	0.2237	0.0489	0.14569	0.343
38	16	1	0.2097	0.0478	0.13411	0.328
39	15	1	0.1957	0.0466	0.12269	0.312
40	13	1	0.1807	0.0454	0.11038	0.296
42	12	2	0.1505	0.0425	0.08653	0.262
43	10	1	0.1355	0.0409	0.07503	0.245
48	9	2	0.1054	0.0369	0.05304	0.209
49	7	2	0.0753	0.0319	0.03278	0.173
50	4	1	0.0565	0.0290	0.02066	0.154
51	3	2	0.0188	0.0181	0.00284	0.125
52	1	1	0.0000	NaN	NA	NA

dose=b

time	n.risk	n.event	survival	std.err	lower 95% CI	upper 95% CI
2	75	1	0.9867	0.0132	0.96105	1.000
3	74	1	0.9733	0.0186	0.93755	1.000
4	73	1	0.9600	0.0226	0.91666	1.000
14	72	1	0.9467	0.0259	0.89716	0.999
15	71	2	0.9200	0.0313	0.86061	0.983
18	69	1	0.9067	0.0336	0.84316	0.975
19	68	1	0.8933	0.0356	0.82613	0.966
20	67	1	0.8800	0.0375	0.80944	0.957
21	66	1	0.8667	0.0393	0.79305	0.947
22	65	3	0.8267	0.0437	0.74529	0.917
23	62	3	0.7867	0.0473	0.69921	0.885
24	59	3	0.7467	0.0502	0.65445	0.852
25	56	3	0.7067	0.0526	0.61079	0.818
26	53	1	0.6933	0.0532	0.59645	0.806
27	52	3	0.6533	0.0550	0.55404	0.770
28	48	4	0.5989	0.0567	0.49743	0.721
29	43	7	0.5014	0.0582	0.39931	0.630
30	36	2	0.4735	0.0582	0.37211	0.603
31	33	2	0.4448	0.0581	0.34432	0.575
32	31	3	0.4018	0.0576	0.30340	0.532
33	28	4	0.3444	0.0561	0.25033	0.474
34	23	1	0.3294	0.0556	0.23667	0.459
37	22	2	0.2995	0.0544	0.20975	0.428
38	20	2	0.2695	0.0529	0.18342	0.396
39	18	1	0.2546	0.0521	0.17048	0.380
40	17	2	0.2246	0.0501	0.14511	0.348
41	15	1	0.2096	0.0489	0.13269	0.331
42	14	1	0.1947	0.0477	0.12047	0.315
44	13	3	0.1497	0.0431	0.08513	0.263
45	10	2	0.1198	0.0394	0.06290	0.228
46	8	2	0.0898	0.0348	0.04209	0.192

47	6	1	0.0749	0.0320	0.03237	0.173
48	5	1	0.0599	0.0289	0.02325	0.154
50	4	2	0.0299	0.0208	0.00767	0.117
51	2	2	0.0000	NaN	NA	NA

dose=c

time	n.risk	n.event	survival	std.err	lower 95% CI	upper 95% CI
2	75	1	0.9867	0.0132	0.9610	1.000
3	74	1	0.9733	0.0186	0.9375	1.000
5	73	2	0.9467	0.0259	0.8972	0.999
6	71	1	0.9333	0.0288	0.8786	0.992
7	70	4	0.8800	0.0375	0.8094	0.957
16	66	2	0.8533	0.0409	0.7769	0.937
17	64	1	0.8400	0.0423	0.7610	0.927
18	63	1	0.8267	0.0437	0.7453	0.917
19	60	2	0.7991	0.0464	0.7132	0.895
20	58	1	0.7853	0.0476	0.6974	0.884
23	57	1	0.7716	0.0487	0.6818	0.873
24	56	4	0.7164	0.0525	0.6207	0.827
25	52	5	0.6476	0.0557	0.5470	0.767
26	47	1	0.6338	0.0562	0.5326	0.754
27	46	6	0.5511	0.0581	0.4482	0.678
28	39	7	0.4522	0.0585	0.3509	0.583
29	32	3	0.4098	0.0579	0.3107	0.541
30	29	5	0.3391	0.0559	0.2455	0.468
31	23	3	0.2949	0.0541	0.2058	0.423
32	20	2	0.2654	0.0526	0.1800	0.391
33	18	3	0.2212	0.0496	0.1425	0.343
41	15	2	0.1917	0.0472	0.1183	0.311
42	13	2	0.1622	0.0443	0.0950	0.277
43	11	2	0.1327	0.0409	0.0726	0.243
46	9	1	0.1180	0.0389	0.0618	0.225
47	8	3	0.0737	0.0316	0.0318	0.171
48	5	5	0.0000	NaN	NA	NA

dose=d

time	n.risk	n.event	survival	std.err	lower 95% CI	upper 95% CI
3	75	1	0.9867	0.0132	0.96105	1.000
7	74	2	0.9600	0.0226	0.91666	1.000
8	72	1	0.9467	0.0259	0.89716	0.999
11	71	1	0.9333	0.0288	0.87855	0.992
12	70	1	0.9200	0.0313	0.86061	0.983
13	69	2	0.8933	0.0356	0.82613	0.966
14	67	2	0.8667	0.0393	0.79305	0.947
15	65	1	0.8533	0.0409	0.77691	0.937
16	64	2	0.8267	0.0437	0.74529	0.917
17	62	5	0.7600	0.0493	0.66924	0.863
18	57	5	0.6933	0.0532	0.59645	0.806
19	52	3	0.6533	0.0550	0.55404	0.770
20	49	1	0.6400	0.0554	0.54009	0.758
21	48	1	0.6267	0.0559	0.52623	0.746
23	47	3	0.5867	0.0569	0.48517	0.709
24	44	4	0.5333	0.0576	0.43158	0.659
25	40	1	0.5200	0.0577	0.41838	0.646
26	39	2	0.4933	0.0577	0.39222	0.621
27	37	3	0.4533	0.0575	0.35358	0.581
28	34	3	0.4133	0.0569	0.31565	0.541
29	31	2	0.3867	0.0562	0.29077	0.514
30	29	2	0.3600	0.0554	0.26623	0.487
31	27	1	0.3467	0.0550	0.25409	0.473
32	26	1	0.3333	0.0544	0.24203	0.459
33	25	3	0.2933	0.0526	0.20645	0.417
36	21	1	0.2794	0.0519	0.19412	0.402
37	20	3	0.2375	0.0494	0.15791	0.357
38	16	4	0.1781	0.0451	0.10840	0.293
39	12	2	0.1484	0.0422	0.08501	0.259
40	10	1	0.1336	0.0405	0.07373	0.242
41	9	1	0.1187	0.0386	0.06276	0.225

42	7	1	0.1018	0.0366	0.05025	0.206
46	6	1	0.0848	0.0342	0.03844	0.187
47	5	3	0.0339	0.0231	0.00894	0.129
48	2	2	0.0000	NaN	NA	NA

dose=e

time	n.risk	n.event	survival	std.err	lower 95% CI	upper 95% CI
3	75	1	0.9867	0.0132	0.96105	1.000
4	74	1	0.9733	0.0186	0.93755	1.000
5	73	1	0.9600	0.0226	0.91666	1.000
6	72	1	0.9467	0.0259	0.89716	0.999
7	71	1	0.9333	0.0288	0.87855	0.992
8	70	1	0.9200	0.0313	0.86061	0.983
10	69	1	0.9067	0.0336	0.84316	0.975
11	68	1	0.8933	0.0356	0.82613	0.966
12	67	1	0.8800	0.0375	0.80944	0.957
13	66	1	0.8667	0.0393	0.79305	0.947
14	65	1	0.8533	0.0409	0.77691	0.937
15	64	2	0.8267	0.0437	0.74529	0.917
16	62	2	0.8000	0.0462	0.71441	0.896
17	59	6	0.7186	0.0521	0.62349	0.828
18	53	4	0.6644	0.0548	0.56530	0.781
19	48	2	0.6367	0.0559	0.53613	0.756
20	46	1	0.6229	0.0563	0.52169	0.744
21	45	1	0.6090	0.0568	0.50736	0.731
22	44	1	0.5952	0.0571	0.49312	0.718
23	43	2	0.5675	0.0577	0.46492	0.693
24	41	1	0.5537	0.0580	0.45097	0.680
25	40	1	0.5398	0.0581	0.43710	0.667
26	39	4	0.4845	0.0584	0.38252	0.614
27	35	3	0.4429	0.0581	0.34251	0.573
28	32	4	0.3876	0.0571	0.29043	0.517
29	28	2	0.3599	0.0562	0.26494	0.489
30	26	1	0.3460	0.0558	0.25234	0.475
32	24	1	0.3316	0.0553	0.23922	0.460
33	23	2	0.3028	0.0541	0.21335	0.430
34	21	3	0.2595	0.0518	0.17550	0.384
35	18	3	0.2163	0.0488	0.13895	0.337
36	15	1	0.2019	0.0476	0.12709	0.321
37	13	1	0.1863	0.0464	0.11432	0.304
39	12	1	0.1708	0.0451	0.10180	0.287
40	11	2	0.1397	0.0419	0.07765	0.252
41	9	1	0.1242	0.0400	0.06606	0.234
42	7	1	0.1065	0.0380	0.05287	0.214
43	6	1	0.0887	0.0356	0.04042	0.195
44	5	1	0.0710	0.0326	0.02885	0.175
45	4	1	0.0532	0.0289	0.01839	0.154
47	3	1	0.0355	0.0241	0.00938	0.134
48	2	1	0.0177	0.0174	0.00260	0.121
49	1	1	0.0000	NaN	NA	NA

dose=f

time	n.risk	n.event	survival	std.err	lower 95% CI	upper 95% CI
3	75	2	0.9733	0.0186	0.93755	1.000
6	72	1	0.9598	0.0227	0.91628	1.000
7	71	1	0.9463	0.0261	0.89645	0.999
8	70	1	0.9328	0.0290	0.87756	0.991
13	69	1	0.9193	0.0316	0.85935	0.983
14	68	2	0.8922	0.0360	0.82438	0.966
15	66	3	0.8517	0.0413	0.77448	0.937
16	63	4	0.7976	0.0467	0.71115	0.895
17	59	3	0.7570	0.0498	0.66540	0.861
18	56	4	0.7030	0.0531	0.60622	0.815
19	51	5	0.6340	0.0561	0.53304	0.754
20	46	1	0.6203	0.0566	0.51871	0.742
21	45	1	0.6065	0.0570	0.50448	0.729
22	44	1	0.5927	0.0573	0.49034	0.716
23	43	2	0.5651	0.0579	0.46235	0.691

```
   24     41       1   0.5513  0.0581      0.44848        0.678
   25     40       1   0.5376  0.0582      0.43471        0.665
   27     38       1   0.5234  0.0584      0.42059        0.651
   28     36       3   0.4798  0.0587      0.37747        0.610
   29     33       3   0.4362  0.0585      0.33531        0.567
   30     30       2   0.4071  0.0581      0.30772        0.539
   31     28       2   0.3780  0.0575      0.28057        0.509
   32     26       3   0.3344  0.0561      0.24069        0.465
   33     23       5   0.2617  0.0525      0.17664        0.388
   34     18       4   0.2035  0.0482      0.12795        0.324
   35     14       3   0.1599  0.0440      0.09331        0.274
   36     11       1   0.1454  0.0423      0.08220        0.257
   37     10       1   0.1309  0.0405      0.07134        0.240
   39      9       1   0.1163  0.0385      0.06078        0.223
   40      8       2   0.0872  0.0339      0.04070        0.187
   41      6       2   0.0582  0.0282      0.02250        0.150
   44      3       1   0.0388  0.0246      0.01120        0.134
   45      2       1   0.0194  0.0184      0.00302        0.125
   46      1       1   0.0000     NaN           NA           NA

> summary(fit)$table
       records n.max n.start events   *rmean *se(rmean) median 0.95LCL 0.95UCL
dose=a      75    75      75     71 29.82225   1.274642     29      27      32
dose=b      75    75      75     71 31.21084   1.283463     30      28      33
dose=c      75    75      75     71 28.20352   1.399090     28      27      30
dose=d      75    75      75     72 26.96811   1.325782     26      23      30
dose=e      75    75      75     70 26.08695   1.370075     26      22      29
dose=f      75    75      75     70 26.08319   1.226083     28      22      32
>
> ## GRAPHS WITH CONF INTERV ###
> ggsurvplot(fit, data=data,
+            pval = TRUE, conf.int = TRUE,
+            fun = "pct",
+            risk.table = TRUE, # Add risk table
+            risk.table.col = "strata", # Change risk table color by treatment
+            size = 1,
+            linetype = "strata",# Change line type by treatment
+            surv.median.line = "hv", # Specify median survival
+            ggtheme = theme_bw(), # Change ggplot2 theme
+            legend.labs=c("0", "40", "50", "60","70","80"),
+            legend = "bottom",
+            xlab = "Time (days)",  xlim = c(1,52),break.time.by = 2,
+            ylab = "Overall survival probability ")
>
>
> ## GRAPHS WITHOUT CONF INTERV ###
>
> ggsurvplot(fit, data=data,
+            pval = FALSE, conf.int = FALSE,
+            fun = "pct",
+            size = 1,
+            linetype = "strata",# Change line type by treatment
+            surv.median.line = "hv", # Specify median survival
+            ggtheme = theme_bw(), # Change ggplot2 theme
+            legend.labs=c("0", "40", "50", "60","70","80"),
+            legend = "bottom",
+            xlab = "Time (days)",  xlim = c(1,52),break.time.by = 2,
+            ylab = "Overall survival probability ")
>
>
> library(multcomp)
> dose<-summary(glht(mod,linfct = mcp(dose="Tukey")))
> summary(dose)

        Simultaneous Tests for General Linear Hypotheses

Multiple Comparisons of Means: Tukey Contrasts
```

```
Fit: coxme(formula = Surv(time, event) ~ dose + (1 | replicate), data = data)

Linear Hypotheses:
              Estimate Std. Error z value Pr(>|z|)
b - a == 0 -0.008944   0.169264  -0.053   1.0000
c - a == 0  0.223304   0.171541   1.302   0.7842
d - a == 0  0.337504   0.171504   1.968   0.3610
e - a == 0  0.385471   0.172362   2.236   0.2210
f - a == 0  0.449230   0.173890   2.583   0.1011
c - b == 0  0.232248   0.169065   1.374   0.7429
d - b == 0  0.346449   0.168679   2.054   0.3119
e - b == 0  0.394416   0.169707   2.324   0.1842
f - b == 0  0.458174   0.170755   2.683   0.0785 .
d - c == 0  0.114201   0.167751   0.681   0.9841
e - c == 0  0.162168   0.168996   0.960   0.9305
f - c == 0  0.225926   0.169933   1.329   0.7686
e - d == 0  0.047967   0.167972   0.286   0.9997
f - d == 0  0.111726   0.168420   0.663   0.9859
f - e == 0  0.063759   0.169442   0.376   0.9990
---
Signif. codes:  0 ‘***’ 0.001 ‘**’ 0.01 ‘*’ 0.05 ‘.’ 0.1 ‘ ’ 1
(Adjusted p values reported -- single-step method)

> plot(summary(dose))
>
```
