# Supplementary material for: Development of the Sterile Insect Technique to control the dengue vector Aedes aegypti (Linnaeus) in Sri Lanka
Source: PLoS One. 2022 Apr 4;17(4):e0265244. doi: 10.1371/journal.pone.0265244 (PMC8979456; doi:10.1371/journal.pone.0265244)
Supplement: Attachment — Submitted filename: Reply to reviewers.docx [file pone.0265244.s006.docx]

**Reply to reviewers**

**Manuscript No:** **PONE-D-21-28806**

We would like to thank all the reviewers for their valuable comments, which contributed to improve the quality of the manuscript. We would like to emphasize that all suggestive changes have been addressed as much as possible. Changes made in the manuscript have been highlighted for your convenience.

- **Journal Requirements**

**Comment 1**

Please ensure that your manuscript meets PLOS ONE's style requirements, including those for file naming. The PLOS ONE style templates can be found at

https://journals.plos.org/plosone/s/file?id=wjVg/PLOSOne_formatting_sample_main_body.pdf and

<https://journals.plos.org/plosone/s/file?id=ba62/PLOSOne_formatting_sample_title_authors_affiliations.pdf>

**Reply**

We have checked the guidelines and the manuscript is in the recommended format.

**Comment 2**

We suggest you thoroughly copyedit your manuscript for language usage, spelling, and grammar. If you do not know anyone who can help you do this, you may wish to consider employing a professional scientific editing service.

**Reply**

The manuscript has now been corrected for language usage, spelling, and grammar**.**

**Comment 3**

The statement 'The *Ae. aegypti* strain used in this experiment originated from eggs collected from the Ragama Public Health Inspector (PHI) division, located within the District of Gampaha, Sri Lanka in 2018' is not sufficient to determine the source of mosquitos in this study. In your Methods section, please provide additional details regarding the source of the mosquito eggs used in your study (if Ragama Public Health Inspector (PHI) division provided the eggs for free or as a donation please provide more information detailing this e.g. if the eggs are part of a insectary colony at the Ragama Public Health Inspector (PHI) division that were donated to this project, or e.g. the eggs are donated from Ragama Public Health Inspector (PHI) division in routine season surveillance of [provide name of area where surveillance occurs]) . If these was collected by members of this study please include geographic coordinates of your field collection site if available and provide the permits you obtained for the work. Please ensure you have included the full name of the authority that approved the field site access and, if no permits were required, a brief statement explaining why. If this was purchased then please include the names of the purchasing sources (e.g., stores, markets, suppliers), if available, as well as any further details about the purchased items (e.g., lot number, source origin, description of appearance) to ensure reproducibility of the analyses. For more information regarding PLOS' policy on materials sharing and reporting, see https://journals.plos.org/plosone/s/materials-and-software-sharing#loc-sharing-materials.

**Reply**

Comment is well taken. The section was amended as follow.

“An adult mosquito surveillance was conducted in the Ragama Public Health Inspector (PHI) division, located within the District of Gampaha, Sri Lanka in 2018 and captured mosquitoes were transported to the laboratory for mass rearing at the Molecular Medicine Unit, Faculty of Medicine, University of Kelaniya, Sri Lanka. Within the laboratory, only the *Ae. aegypti* mosquitoes were separated through morphological identification by well-trained entomologists. Eggs laid by a single *Ae. aegypti* blood-engorged female were used to establish a mosquito colony of *Ae. aegypti.* Each colony was maintained in 24 x 24 x 24 cm cages with mesh screening on top, under a 12:12 (light:dark) cycle at standard conditions (at 27 ± 2 ∘C and 75 ± 5% humidity)”.

**Comment 4**

Please include your tables as part of your main manuscript and remove the individual files. Please note that supplementary tables (should remain/ be uploaded) as separate "supporting information" files.

Please review your reference list to ensure that it is complete and correct. If you have cited papers that have been retracted, please include the rationale for doing so in the manuscript text, or remove these references and replace them with relevant current references. Any changes to the reference list should be mentioned in the rebuttal letter that accompanies your revised manuscript. If you need to cite a retracted article, indicate the article’s retracted status in the References list and also include a citation and full reference for the retraction notice.

**Reply**

Point is well taken. The tables were included as part of our main manuscript. The references list was reviewed.

- **Reviewer 1**

**Comment 1**

Authors affiliations:

4"Insect Pest Control Laboratory" instead "Insect Pest Control Subprogramme"

**Reply**

Sorry but by per co-author instructions, we need to keep Subprogramme since this is the official contact for the IAEA lab.

**Comment 2**

Abstract

Delete the space before the full-stop at the end of the 1st sentence ‘after *Ae. Aegypti’*.

**Reply**

Thank you for your comment. The space was deleted.

**Comment 3**

Materials and methods

Effects of irradiation on flight ability

Remove the “.” after “9 cm diameter” in the sentence “…..150 pupae of one sex were placed in a 9 cm diameter. Petri dish into which a transparent tube…”

**Reply**

OK, the sentence was amended as follow:

“To determine the flight ability of adults, 150 pupae of one sex were placed in a 9 cm diameter Petri dish into which a transparent tube (25 cm in height and 8 cm in diameter) was introduced”.

**Comment 4**

Statistical analysis

Why “the egg hatch rates were arcsinsqrt-transformed prior to analysis”?

This means that the data was not normally distributed? If the case please precise.

**Reply**

The data were not normally distributed and so we arcsinsqrt-transformed to meet requirement for analysis. It has been now mentioned at the statistical analysis section. Thank you.

**Comment 5**

To assess the effects of irradiation on the different parameters (mortality, productivity), the author limits the number of replications to three, however is such studies more replicates are needed to have solid results.

Flies were not available in large number?

**Reply**

Thank you for your comment. In this experiment 150 pupae/dose/sex were considered and repeated 3 times. We could not do more unfortunately due to the number required even for 3 replicates/dose/sex.

**Comment 6**

Mosquitoes -like many insects used for the mass-rearing for SIT- need to be checked for their quality control before using it for SIT. There are several quality control indicators that normally use for this purpose i.e. Mortality/survival, productivity, emergence rate, flight ability, mating competitiveness and insemination rate. In this study, to assess the sterile male mating performance in semi field cage, the author ignore some indicators as the propensity of mating, relative mating index and relative mating performance and limit the study to the competitiveness index.

Or with mosquitoes, is it difficult to evaluate these performance indicators?

Reply

In this study, we decided to focus on competitiveness, because it is the most important quality control indicator for SIT trials, even if others can be used as well, see Bouyer et al. (2020) (Bouyer, Jérémy, and Marc JB Vreysen. "Yes, irradiated sterile male mosquitoes can be sexually competitive!." *Trends in Parasitology* (2020).

.

**Comment 7**

What means “Hn” and “Hs” in the Friend formula “(Hn-Ho) / (Ho-Hs))*(N/S)”? Please define.

**Reply**

We welcome the point. The following section was included in the manuscript to address the above comment.

“The formula (Hn-Ho) / (Ho-Hs))*(N/S) was used to calculate the Fried Competitiveness index (C), where Hn and Hs denotes hatch rate from eggs of females mated with untreated and sterile males, respectively, Ho is the observed egg hatch rate when sterile and wild males are competing, N is the number of untreated males and S is the number of sterile males”

**Comment 8**

The author needs to submit the data analysis details in Rmarkdown file to make the follow up of the data is clear and simple.

**Reply**

All data files were uploaded.

**Comment 9**

Results

1. The figure 2 must be announced in the “Effects of irradiation on flight ability”

**Reply**

OK, the figure 2 was announced at the ‘Effects of irradiation on flight ability’ section. Thank you.

**Comment 10**

In the section “Effects of irradiation on adult survival”, please cheek the figure numbers

**Reply**

OK, figure numbers were corrected in the section “Effects of irradiation on adult survival”,

**Comment 11**

Section “Competitiveness index (C) of sterile male mosquitoes”:

- The tables 1 and 2 are announced but the tables are missing in the document.

Reply

OK, sorry for that, Tables 1 and 2 were included into the document.

**Comment 12**

The author took about 5:1:1 ratio in the result section but is not mentioned in the methodology section

**Reply**

OK, the corresponding section was amended as follow.

Laboratory conditions

Four to five days old sterilized and untreated males were introduced into adult mosquito cages (30 cm × 30 cm × 30 cm) together with virgin females of the same age at the 1:1:1, 3:1:1 and 5:1:1 ratios (sterile males: untreated males: virgin females) (n=100). Furthermore, 1:1 ratio of sterile males: virgin females and 1:1 ratio of untreated males: virgin females were introduced into separate cages as sterile and fertile controls. Adult mosquitoes were fed with cattle blood and fed females were counted. Blood-fed females were allowed to lay eggs and the numbers of eggs laid in each egg cup were counted. Collected eggs were hatched and larvae were counted.

Semi-field conditions

Two experimental ratios were tested in semi-field cages (1.82 m × 1.21 m × 1.21 m) after 4 to 5 days of emergence; sterile males: fertile males: virgin females 1:1:1, 3:1:1 and 5:1 (n=100). Controls were set as 1:1 ratio of sterile males: virgin females and 1:1 ratio of untreated males: virgin females. After a mating period of three days, females were collected into cages (30 cm × 30 cm × 30 cm) and brought to the laboratory. Mosquitoes were blood fed and egg hatch rates were compared as done previously.

- **Reviewer 2**

**Comment 1**

The manuscript shows a very encouraging beginning of SIT development for Sri Lanka. Great job of the IAEA supporting with technical expertise for this country scientific group. Overall, each of every section in the manuscript accomplishes with academic rules of experimental design; i.e, sample size, repetitions, lab rearing conditions, etc. Field research of mark-release-recapture should be started as soon as possible in order to settle preliminary releases of sterile males in Sri Lanka.

**Reply**

Authors are thankful for the comment. Field research of mark-release-recapture were completed now and manuscript for mark-release-recapture is being prepared.
